# Compulsory Vaccination

**Published:** 1866-07

**Authors:** 


COMPULSORY VACCINATION.

Compulsory Vaccination was presented as a subject of discussion by Dr. A. N. Bell, chairman of the committee on that subject. While he regarded the literature of *vaccination for the profession* exhausted, he considered the field still large for the purposes of the committee, namely, the diffusion of information among the people; and his chief object in presenting the subject in this manner, instead of by formal report, was to elicit the expression of the Section as to the best means of diffusing information. In the Southern States, especially, a new field was now open for reaching a large number of persons requiring vaccination and revaccination, in order to put a stop to the ravages of small-pox, and he hoped that some of the gentlemen present would be able to assist the Committee in devising means for such a purpose.

Dr. Wm. M. Charters, of Georgia, described the terrible ravages of small-pox among the negroes at Savannah on the arrival of General Sherman's army, and attributed it, not to any want of a correct appreciation of the utility of vaccination, but to the *imperfect manner* in which vaccination was performed.

It was the practice, to a great extent, to commit this subject to unprofessional persons. In the Southern States, especially, masters and overseers thought themselves equally competent with physicians for so simple an operation. But the result justified the conclusion that the disease they communicated was frequently a *vaccinoid* sore only, and not a vaccine vesicle. The operation was performed more with reference to making a *sore* than anything else, and that was regarded as the prophylactic, but exposure proved to the contrary. In other cases, however, and these were by no means insignificant, vaccination had been well performed and it had taken perfectly, but the susceptibility to it and to small-pox not having been exhausted, the individual was was still liable. He therefore advocated exhaustive vaccination to begin with; let the operation be repeated until it ceases to produce an impression, and he believed susceptibility to small-pox would be destroyed for the whole lifetime of the individual. To accomplish this, he believed in the necessity of compulsory laws, requiring every person to be thoroughly vaccinated, under such penalties as would insure compliance.

Drs. Lee and Hibberd referred to the ineffectual State laws on the subject, and also to the no less ineffectual laws of England, and thought that something more was required.

Dr. Nebinger did not believe that such laws could ever be made effectual in this country, and thought that the work of the Committee to diffuse information—which, so far as vaccination without danger, or a belief in its prophylactic powers was concerned was effectual—only now need to be pressed to the equal necessity of *re*vaccination. On the necessity of *re*vaccination, he knew that there were yet many physicians and a multitude of other people needing information. He cited several cases in illustration. He hoped that the Committee would be continued, and that every member of the Section would do his utmost to press upon the attention of the profession and the public the facts that had been already presented in the last year's report.

Dr. Toner expected much good to result from registration, a subject that was now attracting much attention; and when it was so far advanced that correct information could be gained as to who was and who was not vaccinated, he thought that one great obstacle would be removed—the necessity of universal vaccination would be so much more apparent that the purpose of the Committee would be much facilitated.

On motion, it was voted that the Section recommend that the Committee be continued.

Dr. C. A. Lee introduced *leakage of gas pipes* as a subject well worthy of the consideration of the Section and of the Association.

On motion it was voted that Dr. J. C. Draper, of New York, be requested to prepare a report for the next session of the Association on the leakage of gas pipes.

*Third Session, May 3.*

